# Obstetric and Neonatal Outcomes Among Women With Recurrent Spontaneous Abortions: An Observational Study From a Tertiary Care Hospital in South India

**DOI:** 10.7759/cureus.94632

**Published:** 2025-10-15

**Authors:** Charumathi K B, Kaavya M

**Affiliations:** 1 Obstetrics and Gynecology, Sree Balaji Medical College and Hospital, Chennai, IND

**Keywords:** india, neonatal morbidity, pregnancy outcome, preterm birth, recurrent miscarriage, spontaneous abortion

## Abstract

Background: Recurrent spontaneous abortion (RSA) is a significant reproductive health concern associated with adverse obstetric and neonatal outcomes in subsequent pregnancies. However, evidence from Indian tertiary care settings remains limited.

Objectives: To study patterns of obstetric and neonatal outcomes among women with prior spontaneous abortions and to examine whether the risk of adverse outcomes increases with the number of previous losses.

Methods: This hospital-based observational study was conducted at a tertiary care center in South India from January 2024 to March 2025. Ninety-five pregnant women with ≥2 previous spontaneous abortions (miscarriages) were enrolled consecutively and followed prospectively. Of these, 88 pregnancies completed follow-up and were included in the final analysis. Participants were stratified into two groups: two prior spontaneous abortions (n = 72) and three or more (n = 23). Data were collected using a pre-tested semi-structured questionnaire. Outcomes included mode of delivery, gestational age, intrauterine growth restriction (IUGR), birth weight, Apgar scores, neonatal intensive care unit (NICU) admission, and neonatal complications. Low birth weight was defined as <2.5 kg, and low Apgar score as <7 at one minute. Associations were tested using chi-square and Fisher’s exact tests, with effect sizes expressed as odds ratios (ORs) and 95% confidence intervals (CIs).

Results: Among the 95 pregnancies enrolled, 88 had known outcomes: 81 live births and seven spontaneous abortions, while seven women remained under antenatal follow-up at study completion. Women with three or more prior spontaneous abortions had higher odds of cesarean delivery (OR = 2.8; 95% CI = 1.1-7.5), recurrent loss (OR = 6.3; 95% CI = 1.1-36.1), and preterm birth (OR = 4.0; 95% CI = 1.1-14.2) compared with those with two losses. Neonates born to these women had higher odds of low birth weight (<2.5 kg; OR = 5.1; 95% CI = 1.1-23.1) and low Apgar scores (<7 at one minute; OR = 9.9; 95% CI = 1.0-96.7). Although NICU admissions and neonatal complications were more frequent in this group, the differences were not statistically significant. Given the modest subgroup size (n = 23 for ≥3 losses), these findings should be interpreted as exploratory.

Conclusion: Women with recurrent spontaneous abortions, particularly those with three or more losses, experience higher odds of adverse obstetric and neonatal outcomes. The results suggest a possible dose-response pattern, underscoring the need for early risk stratification, preconception counseling, and intensified antenatal surveillance in this high-risk population.

## Introduction

Spontaneous abortion, or miscarriage, defined as the loss of a clinically recognized pregnancy before fetal viability--operationally <20 weeks of gestation or fetal weight < 500 g, occurring without medical or surgical intervention--remains a major reproductive health challenge worldwide [1]. The World Health Organization estimates that 10-15% of clinically confirmed pregnancies end in miscarriage, though the actual rate is likely higher due to under-reporting of very early losses. Broadly, fetal death before 28 weeks is classified as miscarriage, whereas deaths occurring at or beyond 28 weeks are categorized as stillbirths. Each year, nearly two million stillbirths occur globally, most of which are considered preventable. Yet, both miscarriages and stillbirths are under-recorded and inconsistently reported-even in high-income settings such as the United Kingdom and the United States-suggesting that the true global burden is substantially underestimated [2].

In India, available data indicate a slightly lower but still significant prevalence. The National Family Health Survey-5 (2019-2021) reported a miscarriage rate of 4.9 % among women aged 15-49 years, with more than 80% of these losses occurring in the first trimester [3]. These figures highlight not only the magnitude of pregnancy loss but also the need to address its consequences for subsequent reproductive outcomes. Beyond their frequency, spontaneous abortions are increasingly recognized as prognostic indicators of later obstetric and neonatal complications. Evidence from systematic reviews and observational cohorts demonstrates that women with recurrent miscarriages are at higher risk of preterm birth, low birth weight, neonatal intensive care unit (NICU) admission, and overall perinatal morbidity [4,5].

Several biological mechanisms plausibly explain these associations. Recurrent pregnancy loss may lead to endometrial and myometrial scarring, cervical insufficiency, defective trophoblastic invasion, placental malperfusion, and immune-mediated inflammation-all of which impair implantation and fetal growth, thereby predisposing to preterm labor and growth restriction [6,7]. Such mechanisms provide a pathophysiological rationale linking prior spontaneous abortions with adverse outcomes in subsequent pregnancies.

Against this background, generating robust, context-specific evidence from Indian tertiary-care centers is both timely and essential. In particular, understanding whether an increasing number of prior spontaneous abortions confers a dose-related escalation in perinatal risk--defined as the likelihood of adverse maternal, fetal, or neonatal outcomes occurring from conception to seven days after birth--has important clinical and policy implications. Recognizing such gradients could inform antenatal risk stratification, optimize resource allocation, and strengthen preconception counseling and surveillance strategies for high-risk pregnancies.

Accordingly, the primary objective of this study was to compare obstetric and neonatal outcomes between women with two prior spontaneous abortions and those with three or more. The secondary objective was to describe the overall pattern of obstetric and neonatal outcomes among women with recurrent spontaneous abortions. We hypothesized that women with three or more previous spontaneous abortions would experience higher rates of adverse obstetric and neonatal outcomes compared with those with two prior losses.

## Materials and methods

This hospital-based observational study was conducted in the Department of Obstetrics and Gynecology at Sree Balaji Medical College and Hospital, Bharath University, Chennai, India--a tertiary care referral center with an annual delivery load of approximately 4500-5000 births, serving both urban and semi-urban populations. The study period extended from January 2024 to March 2025. The study population comprised pregnant women attending the antenatal outpatient department, emergency services, or obstetric wards during the period of data collection.

Sample size and sampling technique

The minimum required sample size was estimated using the standard formula for frequency estimation in a population: n = Z2pq/d2, where Z = 1.96 for 95% confidence, p = 0.5 (assumed prevalence of adverse outcomes), and d = 0.1 (absolute precision). The minimum sample size calculated was 88. Allowing for an anticipated 10% non-response rate, the adjusted sample size was 97.6, rounded to 95 for operational feasibility within the 15-month study window. A consecutive sampling method was used, enrolling every eligible participant presenting during the period. We acknowledge that consecutive sampling is non-random and may introduce selection bias; this limitation has been stated in the Discussion. Written informed consent was obtained from all participants.

Inclusion and exclusion criteria

Inclusion Criteria

Pregnant women aged ≥18 years, and those with a history of two or more spontaneous abortions (miscarriages) in previous pregnancies, irrespective of gestational age at loss.

Exclusion Criteria

History of induced or elective abortion, History of spontaneous abortion in multiple gestations, and Presence of multiple gestation in the current pregnancy.

Study tool and pre-testing

Data were collected using a pre-tested, semi-structured proforma developed by the investigators. The proforma captured socio-demographic details, obstetric and medical history, and outcomes of the current pregnancy. The tool was pre-tested among 10 antenatal women with comparable characteristics to assess clarity, relevance, and internal consistency. Minor modifications were made for sequencing and terminology. The reliability of the instrument, assessed using Cronbach's alpha, was 0.83, indicating good internal consistency. Face validity was confirmed through expert review by three senior obstetricians.

Data collection and quality assurance

Data collection was performed by postgraduate residents in Obstetrics and Gynecology, not part of the guiding or analytical team, after standardized training on data collection, ethical practices, and confidentiality procedures. All instruments (e.g., weighing scales) were calibrated daily. Ultrasonography was performed by certified radiologists who were blinded to the study hypotheses. Completed proformas were checked on the same day by the principal investigator for completeness and internal consistency. Data entry was double-verified, with 10% of records cross-checked randomly each week to ensure accuracy and minimize entry errors.

Outcome measures

All participants received routine antenatal care as per institutional protocol. Maternal outcomes included: recurrence of spontaneous abortion, gestational diabetes mellitus, preeclampsia, intrauterine growth restriction (IUGR), malpresentation, placenta previa, and mode of delivery. Neonatal outcomes included: gestational age at delivery, low birth weight (<2.5 kg), low Apgar score (<7 at one minute), need for NICU admission, and neonatal complications (defined as any occurrence of respiratory distress, sepsis, hypoglycemia, or pathological jaundice requiring treatment).

Ethical considerations

The study was approved by the Institutional Human Ethics Committee of Sree Balaji Medical College (Approval No. 002/SBMCH/IHEC/2024/2132) and conducted in accordance with the Declaration of Helsinki. Participation was voluntary, with the right to withdraw at any point without compromising clinical care. Confidentiality was maintained throughout.

Statistical analysis

Data were entered in Microsoft Excel and analyzed using STATA version 14 (StataCorp, Texas, USA). Continuous variables were summarized as means ± standard deviations, and categorical variables as frequencies and percentages. Participants were stratified into two groups based on reproductive history: women with two prior spontaneous abortions and those with three or more.

Associations between abortion history and categorical outcomes were assessed using the Chi-square test or Fisher’s exact test, while independent-samples t-tests were used for continuous variables. A two-sided p < 0.05 was considered statistically significant.

Given the modest sample size and small number of outcome events, multivariate logistic regression was not performed, as it would not meet the minimum event-per-variable (EPV) requirement and could yield unstable estimates. Therefore, results are interpreted as exploratory, emphasizing the observed direction and pattern of associations rather than causal inference.

## Results

A total of 95 pregnant women with a history of two or more spontaneous abortions were enrolled. Of these, 88 pregnancies had known outcomes, comprising 81 live births and seven spontaneous abortions, while seven women remained under antenatal follow-up at the time of study completion. No stillbirths or intrauterine deaths occurred among the live births. All subsequent analyses are therefore based on 88 pregnancies with known outcomes, and neonatal analyses are restricted to 81 live births. The denominators used for each analysis are clearly indicated in the respective tables.

Women with three or more prior spontaneous abortions exhibited a higher frequency of adverse obstetric outcomes compared with those who had two previous losses. Specifically, the ≥3-loss group showed greater proportions of cesarean delivery, recurrent abortion, and preterm birth, whereas intrauterine growth restriction (IUGR) was slightly more frequent but not statistically significant (Table 1).

**Table 1:** Obstetric outcomes by number of previous spontaneous abortions (n = 88 pregnancies with known outcomes). Analyses restricted to 88 pregnancies with known outcomes. CI: confidence interval; IUGR: intrauterine growth restriction; FGU: fetal growth restriction.

Outcome	Two abortions n = 72% (95% CI)	≥3 Abortions n = 23% (95% CI)	p-value
Cesarean delivery	40.3 (29.3–52.3)	65.2 (42.7–82.8)	0.034
Normal vaginal delivery	47.2 (35.4–59.3)	26.1 (10.2–48.4)	0.041
Assisted vaginal delivery	6.9 (2.6–14.4)	4.3 (0.1–21.9)	0.621
Recurrent spontaneous abortion	2.8 (0.3–9.6)	21.7 (7.5–43.7)	0.008
Preterm birth (<37 weeks)	9.7 (4.2–18.3)	26.1 (10.2–48.4)	0.041
IUGR/FGR	7.0 (2.6–14.4)	8.7 (1.1–28.0)	0.579

Among neonatal outcomes (Table 2), most infants had normal birth weight and satisfactory Apgar scores. Nevertheless, those born to women with ≥3 prior spontaneous abortions demonstrated markedly higher proportions of low birth weight and low Apgar scores, both statistically significant. Although rates of NICU admission and neonatal complications were also higher in this group, these associations did not reach significance.

**Table 2:** Neonatal outcomes by number of previous spontaneous abortions (n = 81 live births). Analyses limited to 81 live births. CI: confidence interval. Neonatal complications are defined as respiratory distress, sepsis, hypoglycemia, or jaundice requiring treatment. NICU: neonatal intensive care unit, RDS: respiratory distress syndrome.

Outcome	Two abortions n = 70% (95% CI)	≥3 Abortions n = 11% (95% CI)	p-value
Low birth weight (<2.5 kg)	7.7 (3.2–15.2)	30.0 (9.9–59.6)	0.028
Low Apgar (<7 at 1 min)	1.1 (0.0–6.2)	10.0 (0.3–44.5)	0.030
NICU admission	20.0 (12.1–30.0)	45.5 (16.7–76.6)	0.082
Neonatal complications (RDS, sepsis, hypoglycemia, jaundice)	20.0 (12.1–30.0)	45.5 (16.7–76.6)	0.117

**Figure 1:**
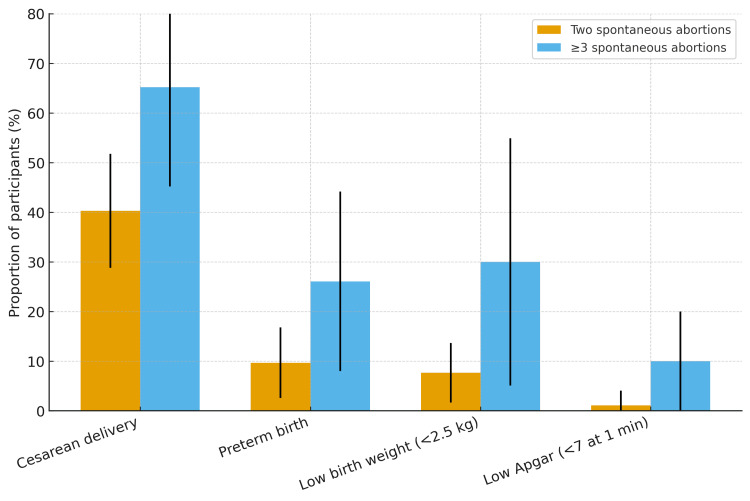
Comparative pattern of adverse obstetric and neonatal outcomes among women with two versus ≥3 prior spontaneous abortions. Bars represent crude proportions with 95% confidence intervals for cesarean delivery, preterm birth, low birth weight (<2.5 kg), and low Apgar score (<7 at one minute).

A comparative summary of obstetric and neonatal outcome proportions, including 95% confidence intervals, is shown in Figure 1, illustrating the consistent upward trend of risk with an increasing number of prior spontaneous abortions.

## Discussion

In this prospective analysis of 95 women with a history of recurrent spontaneous abortions, we found that although the majority of pregnancies resulted in live births, these women continued to experience substantially elevated risks of adverse obstetric and neonatal outcomes. Nearly equal proportions delivered by normal vaginal and cesarean section, with a small proportion requiring assisted vaginal delivery.

Importantly, women with three or more prior losses demonstrated higher frequencies of cesarean section, recurrent abortion, and preterm birth compared with those with only two losses. Correspondingly, neonatal outcomes revealed higher proportions of low birth weight, reduced Apgar scores, and increased NICU admissions among these women, underscoring the cumulative adverse effect of repeated pregnancy loss on perinatal health.

Comparison with existing literature

Our findings are consistent with prior international and regional studies that have identified recurrent pregnancy loss (RPL) as a significant predictor of adverse obstetric and neonatal outcomes. Ausbeck et al. (2019) [8] reported increased risks of gestational diabetes and earlier mean gestational age among women with recurrent miscarriage, while Yang et al. (2017) [9] demonstrated higher cesarean and placental complication rates. Similarly, Sun et al. (2024) [10] found that women with ≥3 miscarriages had greater odds of very low birth weight and preterm birth. The pattern observed in our study is suggestive of a dose-related association, wherein increasing numbers of prior losses may correspond to heightened obstetric and perinatal vulnerability. However, given our modest sample size and lack of multivariate adjustment, these findings should be interpreted as associations rather than causal relationships.

Indian evidence, such as that by Vijayalakshmi et al. (2023) [11], parallels our results, reporting elevated risks of preterm birth, cesarean section, and low birth weight among women with a history of spontaneous abortion. Discrepancies in magnitude between our findings and those of larger multicentric cohorts may reflect differences in population structure, antenatal care access, nutritional status, and definitions of preterm birth. Biological mechanisms plausibly linking recurrent miscarriage to adverse perinatal outcomes include defective trophoblastic invasion, endometrial inflammation, immune dysregulation, uterine scarring, and vascular malperfusion, all of which compromise placentation and fetal growth [12]. These pathophysiological pathways have been well described in ESHRE and RCOG guidelines, reinforcing their global relevance to reproductive risk counseling.

Notably, our study extends this evidence base to a South Indian tertiary-care context, where socioeconomic disparities, undernutrition, and limited early antenatal surveillance may amplify these biological risks. Similar context-specific findings were noted (2021) [13] and emphasized the interaction between maternal anemia, nutritional deficiencies, and poor perinatal outcomes among women with prior miscarriages. Consideration of unmeasured confounders, including maternal comorbidities (e.g., thyroid disease, hypertension), BMI, and socioeconomic status, is essential, as these factors likely contribute to the observed associations.

Critical appraisal and integrated limitations

While the observed trends support the plausibility of a cumulative risk pattern, certain methodological limitations must temper interpretation. The cross-sectional analytic framework precludes causal inference, and the absence of a control group of women without abortion history restricts comparative adjustment. Recall bias may have influenced the reporting of prior losses, though records were cross-verified wherever possible. The small sample size and single-center nature of this study may limit generalizability and reduce statistical power, particularly for rarer neonatal complications. Furthermore, multivariate logistic regression could not be performed due to limited event counts, which restricts our ability to control for confounders. These constraints should be viewed in conjunction with the study’s strengths, including prospective data collection and uniform antenatal care protocols.

Clinical and public health implications

Our findings highlight the need for early identification and intensified surveillance of women with recurrent spontaneous abortions. Classifying such women as a distinct high-risk obstetric group can guide targeted resource allocation. Interventions should include: early booking and individualized antenatal follow-up with serial fetal growth monitoring; screening for modifiable maternal factors such as anemia, nutritional deficiencies, thyroid dysfunction, and short interpregnancy intervals; psychological support and counseling, as recurrent loss can induce grief, anxiety, and depression requiring professional and family-based support [14]; and multidisciplinary coordination among obstetricians, endocrinologists, neonatologists, nutritionists, and psychologists. Strengthening antenatal care protocols in line with ESHRE and RCOG recommendations [15,16] could mitigate preventable perinatal morbidity. Additionally, community-level health education and improved preconception nutrition programs are crucial for reducing recurrence risk and enhancing maternal well-being.

Strengths and limitations

This study’s major strengths lie in its focus on a well-defined, clinically high-risk population and the prospective design, which minimized data incompleteness. The stratification by number of prior abortions allowed identification of a trend suggestive of increasing risk with additional losses. However, limitations include sample size, the absence of a control group, recall bias, and the lack of multivariate analysis, all of which constrain causal interpretation. Hospital-based recruitment further limits external validity to primary care or community settings.

## Conclusions

This study adds novel, context-specific evidence that women with recurrent spontaneous abortions-particularly those with three or more prior losses-face significantly higher risks of adverse obstetric and neonatal outcomes, including preterm birth, cesarean delivery, low birth weight, and reduced Apgar scores. The observed association suggests a possible dose-related pattern, but should not be construed as causal. These findings underscore the cumulative impact of repeated pregnancy loss on maternal and fetal health and call for integrated preconception and antenatal risk-stratification protocols. Strengthening psychosocial, nutritional, and multidisciplinary care strategies will be essential to mitigate preventable morbidity and improve perinatal outcomes among this high-risk group.

## Appendices

Section A: Socio-Demographic Profile

Age (in years): ___

Residence:

□ Urban

□ Semi-urban

□ Rural

Section B: Obstetric & Gynecological History

Gravida: ___

Para: ___

Number of live births: ___

Number of spontaneous abortions: ___

Gestational age of each loss (if known): ___

Any induced abortion? □ Yes □ No

History of multiple gestations? □ Yes □ No

Interval since last pregnancy (in months): ___

History of infertility treatment? □ Yes □ No

Section C: Medical & Surgical History

History of chronic illnesses:

□ Hypertension

□ Diabetes mellitus

□ Thyroid disorder

□ Others (specify): ___

Section D: Current Pregnancy

Gestational age at booking (weeks): ___

Antenatal care initiation:

□ 1st trimester

□ 2nd trimester

□ 3rd trimester

Complications during pregnancy:

□ Gestational diabetes

□ Preeclampsia

□ Placenta previa

□ Malpresentation

□ Intrauterine growth restriction (IUGR)

□ Others: ___

Number of antenatal visits attended: ___

Ultrasonography findings: ___

Section E: Delivery & Maternal Outcome

Mode of delivery:

□ Normal vaginal delivery

□ Assisted vaginal delivery

□ Cesarean section

Gestational age at delivery: ___ weeks

Any recurrence of abortion in current pregnancy? □ Yes □ No

Section F: Neonatal Outcome

Birth weight: ___ g

□ ≥2.5 kg (Normal)

□ <2.5 kg (Low birth weight)

Apgar score:

1 minute: ___

5 minutes: ___

NICU admission required? □ Yes □ No If yes, duration (days): ___

Neonatal complications:

□ Respiratory distress

□ Sepsis

□ Hypoglycemia

□ Jaundice

□ Others: ___

Neonatal outcome:

□ Alive

□ Abortion (<24 weeks)

□ Stillbirth

□ Intrauterine death
